# Interplay between Cell Death and Cell Proliferation Reveals New Strategies for Cancer Therapy

**DOI:** 10.3390/ijms23094723

**Published:** 2022-04-25

**Authors:** Luke V. Loftus, Sarah R. Amend, Kenneth J. Pienta

**Affiliations:** 1 Cellular and Molecular Medicine Program, Johns Hopkins School of Medicine, Baltimore, MD 21205, USA; samend2@jhmi.edu (S.R.A.); kpienta1@jhmi.edu (K.J.P.); 2 The Brady Urological Institute, Johns Hopkins School of Medicine, Baltimore, MD 21287, USA

**Keywords:** cell death, cancer resistance, apoptosis, immunogenic cell death, chemotherapy, cancer cell life-cycle

## Abstract

Cell division and cell death are fundamental processes governing growth and development across the tree of life. This relationship represents an evolutionary link between cell cycle and cell death programs that is present in all cells. Cancer is characterized by aberrant regulation of both, leading to unchecked proliferation and replicative immortality. Conventional anti-cancer therapeutic strategies take advantage of the proliferative dependency of cancer yet, in doing so, are triggering apoptosis, a death pathway to which cancer is inherently resistant. A thorough understanding of how therapeutics kill cancer cells is needed to develop novel, more durable treatment strategies. While cancer evolves cell-intrinsic resistance to physiological cell death pathways, there are opportunities for cell cycle agnostic forms of cell death, for example, necroptosis or ferroptosis. Furthermore, cell cycle independent death programs are immunogenic, potentially licensing host immunity for additional antitumor activity. Identifying cell cycle independent vulnerabilities of cancer is critical for developing alternative strategies that can overcome therapeutic resistance.

## 1. Introduction

Molecular programs controlling cellular growth, proliferation, and death are highly conserved and lie at the foundation of life. Proliferation proceeds through the cell cycle, a series of coordinated events for cell growth, replication of DNA and organelles, and division into two daughter cells. Single-celled organisms reproduce by a single duplication. Multicellular organisms tightly regulate the cell cycle to support cell production and maintain tissue homeostasis. In both cases, intentional cell death is tied to cell proliferation to ensure productive development and organism fitness. For example, programmed cell death in multicellular organisms is required to define limbs and tissues, prevent the spread of infection, and refine biological systems. Single-celled organisms utilize cell (and thus organism) death for the selection of the fittest, resulting in optimal control of cell numbers. This relationship between cell proliferation and cell death leads to the intriguing scientific theory that these are evolutionarily linked opposing processes essential to growth and development [1].

In accordance with their fundamental importance, dysregulation of either cell proliferation or cell death is a driver, or at least a defining characteristic, of numerous diseases such as autoimmunity, neurodegenerative disorders, and cancer. Cancer, in particular, exemplifies the consequences of both unbalanced proliferation and death, whereby unchecked growth rate (e.g., sustaining proliferative signaling, evading growth suppressors) and decreased cell death (e.g., resisting cell death, avoiding immune destruction) leads to aberrant growth. Cancer’s principle imbalance is defined by the Hallmarks of Cancer and further enables an additional proposed Hallmark of Lethal Cancer, therapeutic resistance [2,3]. As cancer and cell death research has progressed, understanding of the underlying biology has evolved. It is now well established that cell death initiated by cell cycle checkpoints proceeds through apoptosis, a molecularly controlled form of cell suicide. Coupled with the knowledge that apoptosis is the primary form of cell death during development, we can clarify that the evolutionary link between cell death and proliferation is between apoptosis and the cell cycle [1,4].

Classical single- and multi-drug therapeutics manipulate the Hallmarks of Cancer by targeting multiple aspects of cell proliferation with the intent of serially eliminating hyperproliferative cells [5]. Modern cancer therapeutics have evolved to include targeting cancer’s manipulation of apoptotic programs and novel antiproliferation modalities. Virtually all of these rely on inducing arrest at cell cycle checkpoints and consequent cell death, i.e., apoptosis. Although apoptosis and the cell cycle are two of the most well-studied pathways in biology, their reciprocal influence is often overlooked. Even less appreciated is that apoptosis eliminates a cell with minimal perturbation of the surrounding tissue and is programmed to proceed without licensing of adaptive immune cells. Taken together, inducing apoptosis alone is an improbable route to reaching comprehensive eradication of cancer.

Beyond apoptosis, there are other mechanisms of regulated cell death specialized in eliminating cells in a variety of contexts. Indeed, cell death is a vital aspect of developmental and homeostatic biology, amounting to a turnover of roughly one percent (around 330 billion) of our cells per day [6,7]. Most of the daily turnover is from cells in direct contact with non-self molecules or in high-stress environments, such as immune cells, gut epithelial cells, and erythrocytes [7]. ***How*** cells die is a critical aspect of maintaining tissue function and homeostasis. In particular, engagement of the immune system distinguishes induced from physiological cell death pathways. Applying this reasoning to cancer reveals an opportunity to reconsider ***how*** therapeutics kill cancer cells. It is known that cytotoxic activity in cancer cells followed by anticancer licensing of the host immune system provides a durable response. However, anticancer strategies rarely consider immunogenic death pathways when attempting to engage host immunity. While there is substantial knowledge of the molecular mechanisms of immunogenic death pathways, their relationship to cancer and proliferation (i.e., the cell cycle) remains underappreciated. A better understanding of how these cell death programs are influenced by proliferation may reveal unknown vulnerabilities in cancer and enable elimination without resistance.

## 2. Immunogenic Death Pathways

The death of individual cells often needs to activate the host immune system to prompt effector cell recruitment and remodeling of damaged tissue. Immune activation following cell death is mainly achieved through a loss of cell membrane integrity and cellular contents released into the extracellular space. One example is passive (primary) necrosis, a type of cell death that occurs from extensive damage to the cell and is independent of signaling pathways. Necrosis can occur from extreme temperature fluctuations, mechanical stress, and high pressures. Secondary necrosis can occur following failure to clear cells that died through nonimmunogenic pathways [8]. Regulated immunogenic death pathways also exist, where molecular events control the elimination of cells following infection, intrinsic damage, elevated reactive oxygen species (ROS) levels, cation imbalance, or innate immune engagement (Table 1). For any death pathway that influences host immunity, signaling pathways need to be tightly controlled to trigger, but not perpetuate, immune functions. If unregulated, immune activation can lead to persistent inflammation, which is considered a core component perpetuating tissue damage in many necrosis-associated diseases like stroke, inflammatory bowel disease, and infectious diseases [9].

### 2.1. Necroptosis

**Figure 1:**
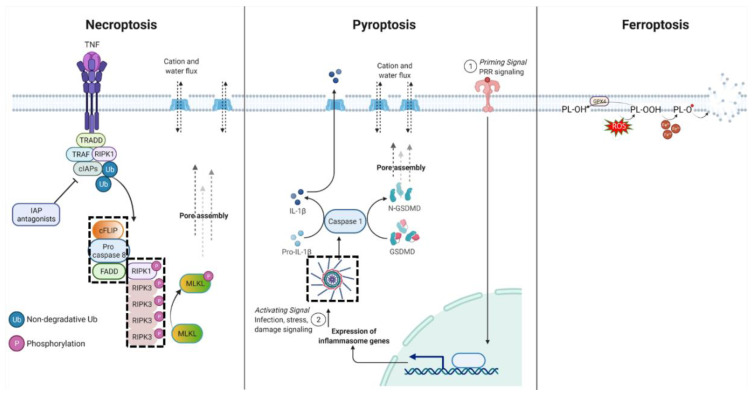
Immunogenic pathways. Dashed box denotes Supramolecular Organizing Centers (see Box 1). Abbreviations: TNF: tumor necrosis factor; TRADD: TNF receptor-associated death domain protein; TRAF: TNF receptor-associated factor; FADD: Fas-associated death domain protein; RIPK1/3: receptor interacting serine threonine kinase 1/3; cFLIP: cellular FLICE-like inhibitory protein; IAP: inhibitor of apoptosis protein family; GSDMD: Gasdermin D; PRR: pattern recognition receptor; PL: phospholipid; GPX4: glutathione peroxidase 4. Created with BioRender.com, accessed on 21 April 2022.

Necroptosis is a molecularly controlled death pathway marked by organelle swelling, pore formation at the membrane, and the release of cellular contents that typically incite a pro-inflammatory response [10] (Figure 1). Necroptotic core components are chiefly implicated in pathophysiological settings as a response to infection and other disease actuators. Additionally, animal studies suggest that necroptosis can compensate for apoptosis deficiencies in development, although clear distinctions from necrosis prevent definitive proof of this phenomenon [11].

Signaling through the tumor necrosis factor superfamily (TNF) and their cognate receptors (TNFRs) initiates necroptosis, with the fate of receptor-interacting serine threonine kinase 1 (RIPK1) being a key molecular event [12]. Ligand engagement by TNFR1 creates a scaffold for the assembly of a membrane-associated complex containing TNFR1 (cytosolic domains), TNFR-associated death domain protein (TRADD), TNFR-associated factor (TRAF)2/5, a cellular inhibitor of apoptosis protein 1/2 (cIAP1/2), and RIPK1 (TNFR Complex I). For necroptosis to be triggered, RIPK1 must exist in a non-ubiquitinated form permitting cytosol trafficking and downstream interactions [13]. This can occur either through the absence of initial ubiquitination by cIAP1/2 (minimal expression or activity) or enzymatic de-ubiquitination (ubiquitin hydrolase CYLD or TNF alpha-induced protein 3). Cytosolic non-ubiquitinated RIPK1 interacts with Fas-associated death domain protein (FADD), TRADD, procaspase-8, and cellular FLICE-like inhibitory protein (cFLIP) to form TNFR Complex IIa held together through interactions between death and death effector domains [13]. Alternatively, through its kinase activity, RIPK1 can spur assembly with RIPK3, cFLIP, and pro-caspase-8 in a complex termed TNFR Complex IIb or the ripoptosome [14,15]; if caspases are active, apoptosis or survival signaling proceeds (discussed later). However, if caspases are sufficiently inhibited, RIPK1 recruits numerous RIPK3 proteins. RIPK1 and RIPK3 trans- and auto-phosphorylate each other facilitating the formation of filaments known as necrosomes. Necrosome formation is thus dependent on initial levels of nonubiquitinated RIPK1, kinase activity of RIPK1 and RIPK3, and adequate inactivation of caspases [13,16]. Active RIPK1/3 activates mixed lineage kinase-like (MLKL) through phosphorylation, which then oligomerizes and associates with the plasma membrane through interactions with cardiolipin and phosphatidylinositol [17,18]. Membrane-associated MLKL influences the formation of additional necrosomes, eventually amplifying MLKL oligomerization, with MLKL oligomers forming pores in the plasma membrane. MLKL pore formation permits the influx of cations, a known event early in necroptotic death [18], but how membrane permeabilization occurs, MLKL pore structure and the actual mechanism(s) of death remain to be definitively elucidated [19]. Notably, necrosome formation can also be initiated through TRIF (adaptor of TLR ligation) and ZBP1 (cytosolic nucleic acid sensor), providing additional methods by which innate immunity can trigger necroptosis.

#### Cell Cycle Implications

TNFR signaling is a central hub capable of driving survival, apoptosis, and necroptosis signaling depending on biological context and the kinetics of initiation. Core proteins are involved in multiple death pathways and serve non-death functions making it challenging to distinguish whether there are direct connections between necroptosis and the cell cycle. The ripoptosome complex, formed downstream of death receptor ligation as well as in response to cellular stress, is particularly relevant as another rheostat guiding survival, apoptosis, and necroptosis fates [15]. By combining pulldown experiments and proximity ligation assays, Liccardi et al. demonstrated that RIPK1:FADD:Caspase-8:cFLIP ripoptosome formation occurred preferentially during mitosis and not in other cell cycle phases, with peak accumulation in metaphase and dissociation following exit from mitosis across various cancer cell lines as well as mouse embryonic fibroblasts [20]. They further demonstrated polo-like kinase 1 (PLK1) and RIPK3 recruitment and association with the ripoptosome. PLK1 (renowned for its role in maintaining genomic integrity by regulation of the G2/M transition and M phase) was recruited through interactions with RIPK1 and cleaved by caspase-8, suggesting tight regulation of PLK1 activity by sequestering in ripoptosome complexes (away from substrates) and cleavage. As outlined above, RIPK3 is a deterministic player in necroptosis membrane rupture but also has apoptotic roles [21]. A recent study clarified the molecular mechanisms at play, showing that PLK1 phosphorylates RIPK3 and protects it from cleavage in the ripoptosome, retaining RIPK3 apoptotic functions during mitosis [22]. These results demonstrate a physiological stacking of apoptosis capacity during mitosis, both independent and dependent on RIPK1/3, likely as a safeguard to preserve genomic integrity during division. Critically, it was proven that RIPK3 associated with RIPK1 retains its necroptotic functions in both G2 and M phases upon direct stimulation and caspase inhibition, but this is necessitated by release from the ripoptosome [22,23]. These studies have important implications for the understanding of cell death during mitosis, genomic integrity, and the main players controlling pathway selection.

It is tempting to draw conclusions about necroptosis susceptibility during mitosis from these experiments. Physiologically there is heavy caspase activity in mitosis to ensure apoptosis capacity as a safeguard of cell division, meaning necroptosis is consequently inhibited. However, in disease or pharmacological settings where caspase activity is inhibited, necroptosis may prevail as a viable backup mechanism to apoptosis during mitosis. This aligns with an analogous theory that necroptosis can serve as a backup to apoptosis in organismal development. However, it must be remembered that necroptosis can proceed without ripoptosome formation through innate immune activators catalyzing direct RIPK1:RIPK3 (necrosome) oligomerization. Additionally, the studies outlined above were performed in highly proliferative cells, mostly cancer, and may not be representative of ripoptosome dynamics in all cell types. Furthermore, necroptosis is efficiently carried out in professional immune cells, including in non- or minorly proliferating states, implying necroptosis competence throughout cell cycle phases or checkpoints [24,25,26]. Overall, necroptosis does not seem to be influenced by cell cycle phase or progression so much as it does by molecular events like caspase activity and intermediates subcellular localization. Follow up experiments on ripoptosome driven necroptosis during and outside of mitosis, ripoptosome independent necroptosis, and non-death functions of the nexus proteins Caspase-8, RIPK1, and RIPK3 will further clarify relationships between necroptosis and the cell cycle (Box 1).

### 2.2. Pyroptosis

Pyroptosis is a lytic form of cell death with concomitant excretion of proinflammatory cytokines to direct immune activation (Figure 1). Cytosolic pattern recognition receptors sense infectious agents and instigate inflammasome formation leading to the caspase- and gasdermin-dependent events of pyroptosis [27]. Inflammasome activation is characterized by a two-step activation process in which a priming signal from damage-associated or pathogen-associated molecular patterns (DAMPs or PAMPs) recognition leads to gene expression of core inflammasome components, caspase-1, pro-IL-18, and pro-IL1B [28]. A second signal comes from the cellular response to a diverse set of microbial, viral, stress, or damage signals that initiate numerous routes to the assembly of a higher-order structure (inflammasome) (Box 1). Large, multimeric inflammasomes recruit numerous procaspase-1 molecules that autoactivate through self-cleavage [28]. Active caspase-1 cleaves gasdermin D (GSDMD), and the N-terminal GSDMD fragments translocate to the membrane, oligomerize, and form multimeric pores in the membrane [29]. As with MLKL oligomerization, GSDMD N-terminal fragments localize to membranes through lipid interactions [30,31]. Caspase-1 also cleaves pro-IL-1B and pro-IL-18 to their mature forms, allowing their exit from the cell via gasdermin pores or following extensive membrane permeabilization. Additionally, caspase-11 can be directly activated by PAMP detection, namely lipopolysaccharide, and contribute to pro-IL-1B and pro-IL-18 cleavage regardless of inflammasome assembly [28]. NLR family pyrin domain containing 3 (NLRP3) is the most studied inflammasome protein and is expressed primarily in myeloid and barrier immune cells demonstrating those cell types’ proficiency in executing pyroptosis and directing immune activation [28,32]. However, pyroptosis may be accessible to a larger range of cell types since there is considerable diversity in inflammasome components and gasdermin isoforms, each with varied expression patterns across cell types. Understanding inflammasome dynamics are complicated by the non-pyroptotic functions of inflammasome monomer proteins, including several that do not participate in pyroptosis [33,34]. Further research is needed on inflammasome proteins to discern their roles before expanding the list of cell types that are capable of undergoing pyroptosis.

#### Cell Cycle Implications

Pyroptosis occurs primarily in cells of the myeloid lineage, which are largely considered to be non-proliferative or lowly proliferative to support self-renewal. Thus, it can be predicted that the initiation and progression of pyroptosis would be agnostic to cell cycle phase or checkpoints. Alternatively, pyroptosis could be tied to a resting cell cycle state, like G0 or G1 stall, but some evidence of pyroptosis in proliferative types makes this hypothesis unfavorable. Acute inflammation following inflammasome activation and pyroptosis has implications for many processes, including proliferation [35,36]. However, these considerations would implicate pyroptosis in influencing the cell cycle as opposed to the cell cycle phase influencing pyroptosis competence, thus lying beyond the scope of this review.

### 2.3. Ferroptosis

Cell death can occur from the accumulation of reactive oxygen species (ROS), leading to unconstrained lipid peroxidation, propagation of radical lipid species, and plasma membrane disruption [37,38]. This death pathway, known as ferroptosis, is distinct from other pathways at the biochemical level based on iron dependence, required lipid involvement, and ROS as the sole initiator (Figure 1). ROS are constitutively produced by physiological processes, in particular metabolic pathways (mitochondrial electron transport chain complexes), and enzymes like nicotinamide adenine dinucleotide phosphate oxidases (NOXs), lipoxygenases (LOXs), and the cytochrome P450 superfamily, with ROS from NOX family enzymes, explicitly involved in the production of lipid ROS species [37,39,40]. When acting upon lipids, ROS remove discrete hydrogen atoms creating lipid radicals that then react with oxygen to form lipid peroxides and propagate the radical [37]. Free iron mediates Fenton reactions that convert lipid hydroperoxides into highly reactive lipid alkoxyl radicals that disrupt membrane integrity and cause ruptures. Iron may also be required for the formation of lipid peroxides by enzymatic (cofactor for LOXL) or non-enzymatic means [41]. Of cellular lipid species, bisallylic hydrogens in polyunsaturated fatty acids (PUFAs) are uniquely susceptible to abstraction due to a lower bind dissociation energy relative to other saturated sites. This is highlighted by data that the rate of lipid peroxidation correlates with unsaturation in membranes and that PUFA generating enzymes are required for ferroptosis to occur [42,43,44]. Ferroptosis is counteracted by a specific member of the glutathione peroxidase family, GPX4, which preferentially reduces large lipid peroxides to nonreactive lipid alcohols, thereby preventing conversion to lipid alkoxyl radicals [38,41]. Some lipophilic small molecule antioxidants, mainly the vitamin E family and ubiquinol-10, can access lipids and thus also protect from lipid peroxidation [39].

#### Cell Cycle Implications

Ferroptosis depends on ROS levels, the abundance of lipid species (preferentially PUFAs), soluble iron, and GPX4 that may be regulated by expression and activity (i.e., glutathione availability). These components have ubiquitous functions in cell physiology, leaving no inclination nor evidence for ties to cell cycle checkpoints or cell cycle progression. Emerging connections between the cell cycle and regulation of cell metabolism are relevant through notably increased de novo lipid synthesis during G2 in preparation for membrane synthesis and division. Interestingly, de novo lipid synthesis has also been shown to be necessary for mitotic exit, primarily for nuclear envelope assembly and/or expansion [45,46,47]. Although an interesting connection between lipid metabolism and mitosis, this does not imply a lack of susceptibility to ferroptosis in other phases of the cell cycle. On the other hand, it is tempting to speculate that cells in a prolonged G2 phase may have elevated lipid content, underlying a heightened vulnerability to ferroptosis induction if ROS levels are also uncontrolled.

### 2.4. Autosis (Autophagy-Dependent)

Autosis is a unique form of cell death reported during starvation, reperfusion injury, and ischemia. Molecular progression relies on autophagic machinery and entails the excessive accumulation of autophagosomes and autolysosomes, separation of nuclear membranes, inflation of the perinuclear space, and eventual loss of organelle and plasma membrane integrity [48,49]. A membrane Na^+^/K^+^ ATPase is crucial for this death pathway as evidenced by inhibitors of Na^+^/K^+^ ATPase, such as cardiac glycosides, capacity to block autosis entirely, although other mediators may also be in play [50]. Further research into the molecular mechanisms of autosis and specific clinical instances beyond cardiac tissue will help elucidate how this form of regulated cell death is employed in physiology and disease states and the role of immunogenic vs. non-immunogenic autophagy-dependent cell death (ADCD).

#### Cell Cycle Implications

No link to the cell cycle (or definitive cell cycle independence) can be proposed for autosis due to the ambiguity of the molecular mechanisms. Furthermore, no conclusions can be drawn between cell cycle machinery and the Na^+^/K^+^ ATPase itself, as the latter is a P-Type ATPase found in all mammalian cells.

Box 1 **Supramolecular Organizing Centers.**

The term Supramolecular Organizing Centers (SMOCs) was coined in 2014 to define higher-order signaling complexes that coordinate cellular innate immune responses [51]. With a focus on structural and dynamic studies, it was described how SMOCs operational advantage comes from increasing local concentrations of otherwise intrinsically weak protein-protein interactions. SMOCs also serve as a common hub to integrate upstream stimuli and direct downstream signaling pathways, providing a powerful method for tying stimulus integration to signaling determination. An explicit example is inflammasomes, which can form from a multitude of proteins, respond to numerous signals, and influence multiple downstream signaling pathways [52]. Biological precedence also exists in complexes such as the proteasome or microtubule organizing center, suggesting that this is a proven modality of cell biology. Originally five SMOCs were described in innate immunity, three of which, the inflammasome (pyroptosis), FAS, DISC, and PIDDosome (apoptosis), control death pathways as well as innate immune signaling [51]. Now we can appreciate that higher-order complexes coordinate many of the death pathways described, such as the apoptosome, ripoptosome, necrosome, MPTP complex, and Nettosomes. Important for death pathways, SMOCs enable signal amplification through cooperative polymerization of nucleated precursors, allowing a relatively small number of sensor proteins to spark SMOC formation, which then recurrently recruits and activates downstream molecules [51,53]. This represents a unique view on signal transduction and is postulated to explain threshold, all-or-none responses seen in innate immunity. Subcellular localization and SMOC degradation to terminate signal transduction are additional considerations that are less elucidated but important for regulation. Despite a compelling Opinion piece in 2014, there has not been significant literature looking at the mechanistic roles of SMOCs outside of innate immunity. Death pathway research has focused on complex formation where necessary, but rarely biochemical dynamics and subcellular localization of signaling hubs. Further research on higher-order complex dynamics will advance understanding of death pathway signal transduction and fill critical gaps in knowledge about inciting one pathway over the other, particularly in regards to temporal and threshold dynamics.

### 2.5. mPTP-Mediated Necrosis

Mitochondrial permeability transition (mPT) refers to acute permeability of the inner mitochondrial membrane, allowing spontaneous ion and water flux into the mitochondrial matrix. mPT is facilitated by a multimeric pore (mPTP), currently believed to consist of adenine nucleotide translocator, the ATP synthase, and cyclophilin D, although pore structure remains to be definitively elucidated [54,55]. Physiologically this pore maintains mitochondrial homeostasis by regulating Ca^2+^ levels and allowing efflux of ROS [56,57]. In disease and stress states, dramatic disruptions to the cytosolic environment, in particular increases in free Ca^2+^, can cause protracted opening of the mPTP. Persistent conductance through the pore leads to respiratory complex disassembly, NADH depletion, increased ROS production, mitochondrial swelling, and dissolution of the membrane potential [55]. Cell death follows if extended mPTP opening occurs throughout the cell through mitochondrial stimulation and escalating susceptibility to mPTP opening. There is considerable evidence for mPTP-mediated cell death being akin to regulated necrosis, likely due to compromised ion gradients and loss of plasma membrane integrity [57,58,59,60]. However, disruptions to mitochondrial membrane integrity also facilitate the release of mitochondrial proteins, including actuators of intrinsic apoptosis (described later). Cellular ATP levels are thought to be one factor governing the advancement of one pathway versus the other (discussed further in Section 3.1), but other factors could be involved as well. Remaining ambiguity on mPTP structure impedes clear distinction between the pore’s role in apoptotic and regulated necrotic death pathways.

#### Cell Cycle Implications

Calcium involvement in many signaling pathways and the lack of clarity in molecular components and mechanisms of mPTP-mediated necrosis prevents hypotheses about susceptibility across the cell cycle.

**Table 1:** Core components of Immunogenic Death Pathways.

	**Necroptosis**	**Pyroptosis**		**Ferroptosis**
Key events	Death Receptor activation inactive caspases MLKL oligomerization	PRR activation inflammasome formation GSDMD oligomerization		ROS accumulation Lipid peroxidation
SMOC	Ripoptosome Necrosome	inflammasome		n/a
effector functions	MLKL pores in the membrane	GSDM pores in the membrane IL-1B and IL-18 release		loss of membrane integrity
cell cycle propensity	no bias; possible backup to apoptosis during mitotic death	no bias; possibly more active in resting phases		no bias
	**Autosis**	**mPTP-Mediated Necrosis**	**Parthanatos**	**NETosis**
Key events	starvation, reperfusion injury N+/K+ ATPase activity	Ca^++^ or ROS imbalance prolonged mPT	PARP1 hyperactivity PAR generation > breakdown MIF activity	ROS accumulation cytoskeletal rearrangements NET extrusion
SMOC	n/a	mPTP complex	n/a	NETTosomes (chromatin, granular proteins)
effector functions	inflated perinuclear space organelle catabolism loss of membrane integrity	loss of mitochondrial gradient plasma membrane rupture	energy depletion DNA fragmentation plasma membrane rupture	NET release loss of plasma membrane integrity
cell cycle propensity	no bias	no bias	likely heightened sensitivity prior to DNA replication	no bias; possibly more active in resting phases

### 2.6. Parthanatos

DNA damage is a prototypical actuator of cell death by either apoptosis (described later) or parthanatos, a death pathway resulting from accumulating activity of the DNA damage response protein poly(ADP-ribose) polymerase 1 (PARP1) [61]. These two pathways work closely together to determine the consequences of cell death.

PARP1 is a well-characterized enzyme of the PARP family most acknowledged for repairing DNA single-strand breaks. Upon DNA damage, PARP-1 adds poly ADP-ribose (PAR) to itself and other substrates with considerable variance in the complexity and length of PAR polymers. PARylated sites recruit and guide DNA repair enzymes to damaged DNA [62]. Excessive or severe DNA damage, however, can lead to PARP-1 hyperactivation, skewing the balance of PAR breakdown vs. PAR generation [63,64]. The buildup of PAR polymers leads to apoptosis-inducing factor (AIF) binding and release from mitochondria, which can occur without concomitant cytochrome c release via mPT. Cytosolic AIF binds macrophage migratory inhibitory factor (MIF), the primary nuclease instigating parthanatos, and promotes MIF nuclear translocation and subsequent DNA fragmentation [63,65]. PARP1 consumes cellular NAD^+^ and ATP stores to accumulate PAR polymers, meaning hyperactivation largely depletes cellular energy, which contributes to cell death. Plasma membrane rupture occurs downstream of parthanatos progression, at least in some settings, making this a form of regulated necrosis [66,67]. NAD^+^ and ATP depletion, as well as incomplete mitochondrial permeabilization, precludes apoptosis progression following hyperactive PARP-1 activity [62]. Alternatively, if excessive DNA damage is sensed before a commitment to DNA repair, then repair would require more energy than is feasible. In this setting, complete mitochondria permeabilization occurs through apoptosis, and caspases cleave PARP1 to prioritize energy usage for apoptotic progression [68]. Thus, parthanatos is a DNA damage-dependent death pathway distinct from apoptosis, and a cell’s response to DNA damage or competence of either pathway dictates decision making [69,70].

#### Cell Cycle Implications

Parthanatos is caused by PARP1 hyperactivity. PARP1 is a multifunctional enzyme that participates in all forms of DNA damage repair, including repair of replication forks and modifying chromatin tertiary structure, primarily as a sensor of DNA damage bridging to corrective machinery. This would mean excessive PARylation and subsequent parthanatos are possible throughout the cell cycle. However, it is worth noting that PARP1 responds rapidly to single-strand breaks and single base modifications and has well-defined interactions with non-homologous end joining (NHEJ) machinery [62]. Both NHEJ and single-strand break correction occur without a template, and the associated enzymes exhibit preferential activity in G1, implying that in DNA damage settings, PARP1 activity may be elevated during G1 [71]. It can be speculated that PARP1 hyperactivity is more susceptible prior to DNA replication as a cell needs to resolve DNA integrity.

### 2.7. NETosis

Neutrophils are vital immune regulators with phagocytic and lytic effector functions for eliminating detrimental pathogens and cells. Another effector function of neutrophils involves the extrusion of chromatin structures bound with granular proteins; a complex termed neutrophil extracellular traps (NETs) [72]. NETs trap and neutralize many foreign pathogens and prevent bacterial and fungal dissemination [72,73,74]. NET release from neutrophils follows nuclear and cytoskeletal structural alterations and can occur with or without lethal plasma membrane disruption, the former being a proven mechanism of neutrophil cell death (NETosis) distinct from apoptosis and necroptosis [72,75,76]. Many physiological stimuli can induce NETosis, but all share ROS accumulation as a driver of NETs formation [77]. NETosis’ impact on physiology and disease is emerging and will further clarify this pathway’s prevalence relative to other death pathways in neutrophils.

#### Cell Cycle Implications

Mature neutrophils are terminally differentiated cells that have lost their proliferative capacity and globally downregulated cyclin and cyclin-dependent kinase (CDK) proteins [78,79]. Despite this, there is evidence that NET production is dependent on some components of cell cycle signaling, particularly CDK4/6 activity. Experiments showed that phosphorylation of retinoblastoma, histones, and lamins occurred with neutrophil activation and NETosis but without DNA synthesis, condensation of chromosomes, or cytokinesis [80]. NETosis capacity was inhibited by p21 mimetics, further proving CDK involvement [80]. Clearly, CDK activity is involved in NET formation, but at present, it is unclear whether or not this is also accompanied by cell cycle progression and checkpoint activity (thus influencing cell death). One hypothesis is that CDK activity is needed solely for nuclear remodeling and not further roles during cell cycle progression to allow NET formation and extrusion.

## 3. Non-Immunogenic Death Pathways

Cell death pathways that do not elicit immune responses involve shrinking and packaging of the target cell, followed by ingestion and degradation by phagocytic cells (Table 2). Successful completion of this entire process is critical for the removal of obsolete, damaged, or infected cells without generating unwarranted inflammation that could be harmful to the local environment or erroneously licenses adaptive immunity. Apoptosis is the most well-recognized of these pathways, though excessive autophagic flux can also escalate to non-immunogenic cell death.

### 3.1. Apoptosis

Apoptosis was first identified in 1972 as a programmed form of cell death that does not elicit immune activation [81]. Cells undergoing apoptosis are characterized by a progressive shrinkage in cell size, cytoskeletal collapse, breakdown of the nuclear envelope, chromatin condensation, and eventual blebbing or budding off of apoptotic cell bodies [82]. Apoptosis can be initiated by several signaling events, all of which converge on caspase-mediated effector functions.

#### 3.1.1. Intrinsic Apoptosis

**Figure 2:**
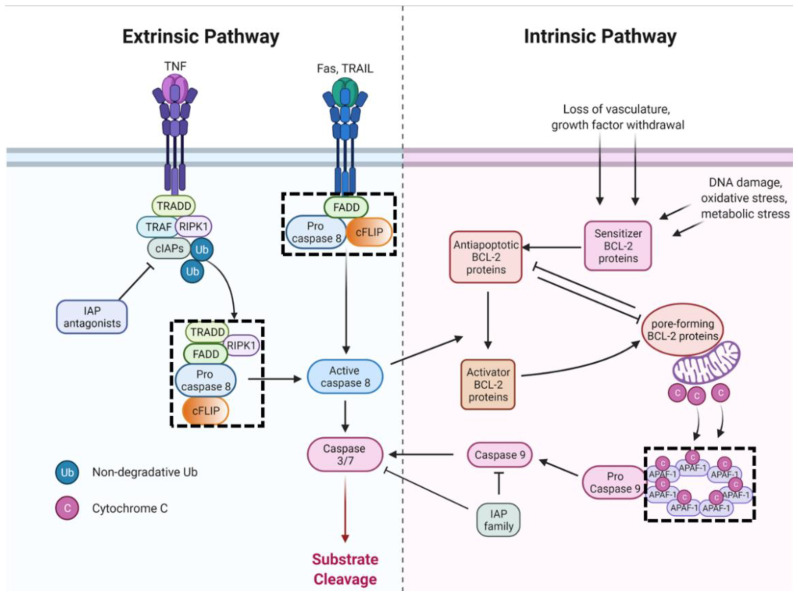
Apoptosis pathways. The dashed box denotes Supramolecular Organizing Centers (see Box 1). Abbreviations: TNF: tumor necrosis factor; TRAIL: TNF-related apoptosis-inducing ligand; TRADD: TNF receptor-associated death domain protein; TRAF: TNF receptor-associated factor; FADD: Fas-associated death domain protein; RIPK1: receptor interacting serine threonine kinase 1; cFLIP: cellular FLICE-like inhibitory protein; APAF-1: apoptotic protease-activating factor 1; IAP: inhibitor of apoptosis protein family. Adapted from “Extrinsic and Intrinsic Apoptosis”, created by BioRender.com, accessed on 21 April 2022.

Intrinsic apoptosis is initiated by a wide variety of developmental and stress stimuli, including lack of growth factors, loss of vasculature, DNA damage, loss of adhesion, metabolic stress, and oxidative stress [4] (Figure 2). Apoptotic responses to death stimuli are mediated by the B cell lymphoma 2 (Bcl-2) family of proteins, a diverse set of proteins that are regulated by their protein-protein interactions, affinity, post-translational modifications, and relative abundance in the mitochondrial membrane [83]. Antiapoptotic, pore-forming, activator, and sensitizer family members coordinate to prevent apoptosis under physiological conditions and initiate mitochondrial outer membrane permeabilization (MOMP) in response to death stimuli or lack of survival signals. MOMP is the central event driving intrinsic apoptosis by enabling the release of downstream proteins [84]. Released cytochrome c binds to cytosolic apoptotic protease-activating factor 1 (APAF1) to form the apoptosome complex, resulting in the recruitment of multiple procaspase-9 proteins. Procaspase-9 forms homodimers and heterodimers with APAF-1, facilitating self-cleavage to mature caspase-9 and dissociation from the apoptosome complex [85]. Active caspase-9, in turn, cleaves (and thereby activates) the effector caspases-3, -6, and -7.

#### 3.1.2. Extrinsic Apoptosis

Apoptosis can also be induced by the engagement of cell surface death receptors to facilitate targeted elimination (Figure 2). Death receptors belong to the TNF superfamily and are distinguished by intracellular death domains (DD) that initiate lethal signaling pathways upon ligand binding [13,86]. Many cell types express low levels of death receptors, while their cognate ligands are primarily expressed by immune cells [87]. The classic molecular mechanism (used by Fas and TNF-related apoptosis-inducing ligand (TRAIL)) involves ligand binding to the death receptor triggering DD association with the adaptor protein FADD. FADD scaffolding recruits procaspase-8, which in turn recruits the inactive caspase-8 homolog cFLIP, forming the Death Inducing Signaling Complex (DISC) [86]. cFLIP and procaspase-8 interactions are master regulation nodes controlling survival, apoptosis, or necroptosis signaling from the DISC. Short isoform cFLIP prevents the recruitment of multiple procaspase-8 proteins, inhibiting apoptosis. The long isoform of cFLIP, when present at low levels, enhance protease activity of procaspase-8:cFLIP heterodimers and promotes oligomerization of procaspase-8 proteins through death effector domain interactions [88,89,90]. Multiple procaspase-8 proteins are then recruited and catalyze self-cleavage to active caspase-8, which is released from the membrane-associated DISC. Activated cytosolic caspase-8 activates effector caspases through proteolytic processing (type I apoptosis) or cleaves the proapoptotic BID protein, which leads to MOMP (type II apoptosis) depending on cell type and molecular context [87].

Death receptor signaling through TNF receptor (TNFR) is more convoluted because TNFR signaling is involved in extrinsic apoptosis, necroptosis, cell survival, and proliferation signaling. Initial ligand engagement by TNFR1 leads to assembly of TNFR Complex I. As with necroptosis, RIPK1 modification status enables downstream signaling, and cooperative dynamics of cFLIP, and procaspase-8 (in TNFR Complex IIa) determine cell fate. Active procaspase-8:cFLIP heterodimers recruit multiple procaspase-8 proteins in proximity, followed by autoproteolytic processing, yielding mature caspase-8 that is released into the cytosol [13,88]. Caspase-8 cleaves effector procaspases to unleash effector caspase activity characteristic of apoptosis. Caspase-8 and active procaspase-8:cFLIP_L_ heterodimers also cleave additional substrates, including procaspase-8 (contributing to activation), RIPK1, and RIPK3 (to inhibit necrotic pathways), and ubiquitin hydrolase CYLD and cFLIP (to control activation through both apoptosis and necroptosis) [88,91,92,93].

#### 3.1.3. Mitotic Death

Abnormal mitosis can occur as a consequence of deficient cell cycle checkpoints, mitotic machinery defects, or failure to sense DNA damage. Resultant cell division is asymmetric, leading to non-diploid daughter cells, which contribute to genetic instability and malignant transformation. In addition to cell cycle mechanisms to prevent unbalanced cell division, cells can also undergo mitotic catastrophe, an active biochemical mechanism that preserves genomic integrity by driving cells to an irreversible state [94]. This fate can be cell death following a prolonged time in mitosis, called mitotic death, or an exit from mitosis and entry into cellular senescence during G1 phase [94,95]. Mitotic death is a regulated form of cell death with distinct induction from canonical intrinsic or extrinsic apoptosis. However, downstream processes are likely aligned with apoptosis as chromatin condensation, the release of cytochrome c and AIF, caspase activity, and DNA degradation all occur following mitotic catastrophe [96]. Proteins linking mitotic catastrophe to apoptosis still need to be fully delineated. There is a consensus that caspase-2 is intimately involved as its activation is an initiating event of mitotic death [95]. Activated caspase-2 contributes to apoptosis by cleaving BID in mitotic death settings, cleaving Golgi proteins, and suppressing the generation of non-diploid cells through p53-dependent and p53-independent mechanisms [95,97].

The alternative outcome of mitotic catastrophe, cellular senescence, is also important in the context of immune cross-talk. Senescent cells often secrete pro-inflammatory cytokines, paracrine factors, and matrix proteases that collectively influence surrounding cells’ proliferative capacity and recruit immune cells. This senescence-associated secretory phenotype (SASP) is particularly relevant in cancer as entry into senescence is a known survival mechanism for chemotherapy, both from mitotic catastrophe and other cell cycle checkpoints [98]. Initially, the SASP is presumably anti-tumorigenic, but when prolonged can aid tumor progression by broadly suppressing local immune activation, promoting neovasculature, and influencing the cell’s migratory and evasive properties [99,100]. Senescence and its characteristics are not a death pathway and thus will not be further discussed, but are additional factors to consider influencing cancer survival and immune cross-talk (reviewed in [99]).

#### 3.1.4. Caspase Activity and Regulation

Effector caspases cleave over 1000 target proteins to kill a cell, including nuclear lamins (irreversible breakdown of nucleus structure); ICAD (frees the DNA endonuclease caspase-activated DNase, CAD, for genomic breakdown); cytoskeletal and adhesion proteins (detachment and rounding); flippase (PS exposure) [82,101]. Additionally, caspases cleave proteins involved in other death pathways, like RIPK1/3, PARP1, Beclin-1, to prevent other forms of cell death from competing with apoptosis. Caspase activation is an irreversible (precursors proteolytically processed to active molecules) and self-amplifying (initiator caspase can cleave multiple effector caspases) cascade that robustly yields cell death. Consequently, under physiological conditions, cells utilize the inhibitor of apoptosis (IAP) protein family to suppress caspase activation and ensure that apoptosis proceeds only when appropriate [82]. IAP proteins function by either binding and inhibiting active caspases or ubiquitinating caspases and thereby marking them for proteasome degradation [102]. Upon death signal stimulation and MOMP, anti-IAP proteins, second mitochondria-derived activator of caspases (SMAC/DIABLO), and Omi are released from the mitochondrial intermembrane space and trigger degradation of IAP proteins [103,104,105]. Cytochrome c combined with SMAC and Omi release allows complete caspase activation and committed execution of apoptosis. Evidence suggests that anti-IAP and cytochrome c release from the mitochondria is temporally regulated as continued activation of pore-forming Bcl-2 members progressively increases the pore size in the outer mitochondria, allowing the escape of cytochrome c then the larger SMAC protein [83].

Another layer of apoptosis regulation occurs through survival factors. Survival factors are often extracellular and bind to cell surface proteins on target cells to inhibit apoptosis through increased production of antiapoptotic Bcl-2 proteins, inactivation of proapoptotic Bcl-2 members, or inactivation of anti-IAPs [82]. Loss of survival factors removes the inhibition of apoptosis and allows cell death to proceed. Examples include cytokine signaling dictating clonal expansion of lymphocytes and apoptosis in epithelial cells following the loss of attachment from the extracellular matrix (termed anoikis) [8].

Additionally, many of the chemical reactions (catabolism of macromolecules, caspase activation) and downstream structural changes (chromatin condensation, apoptosome formation) of apoptosis require ATP, meaning apoptosis is an energy-dependent process [106,107,108]. Several groups have hypothesized that cellular ATP levels constitute an additional determinant of cell death, whereby apoptosis or necrosis is determined by sufficient or insufficient ATP levels, respectively [106,108,109]. There is direct evidence for this phenomenon in settings with human T cells, epithelial cells, and a variety of cancer cell lines [106,107,108,110,111]. Furthermore, ATP levels increase in the early stages of apoptosis and may decrease with apoptotic progression, at least partially due to caspase cleavage of an ATP plasma membrane channel [112,113]. Together this body of work proposes an intriguing concept that energy levels on a cell to cell basis influence apoptosis competence and that regulation of cytosolic ATP are part of the apoptotic program. If ATP levels impede apoptosis events, then necrotic programs can take place instead (again suggesting necrosis, regulated or passive, can function as a backup to apoptosis).

#### 3.1.5. Cell Cycle Implications

**Figure 3:**
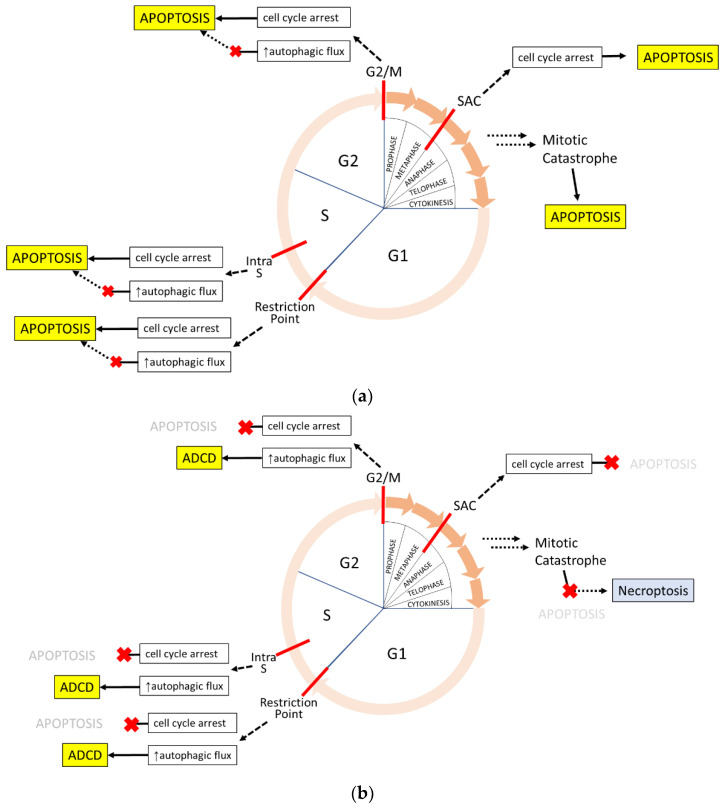
Cell cycle-mediated cell death occurs through non-immunogenic death pathways. (**a**) Cyclin-dependent kinase (CdK) inhibitors act in response to many stressors to arrest the cell cycle and increase autophagic flux. When damage cannot be resolved, autophagy is terminated and the cell commits to apoptosis. Cell death from mitotic catastrophe also proceeds through apoptosis. (**b**) When the apoptosis pathway is compromised or inhibited, as is commonly seen in cancer, cell death may occur through other pathways. Prolonged cell cycle arrest can lead to sustained autophagy and presumably lead to autophagy-dependent cell death (ADCD). During a mitotic catastrophe, spontaneous ripoptosome formation could enable necroptosis without TNF signaling. Blue death programs are immunogenic; yellow are non-immunogenic.

Cell cycle mechanisms have been linked to apoptotic cell death since apoptosis was first described in literature when it was speculated that ‘hyperplasia might sometimes result from decreased apoptosis rather than increased mitosis’ [81]. Observations that mitosis and apoptosis share morphological features such as rounding, nuclear and cell size condensation, and detachment further support integrated pathways [114]. It is now well-known that cell death from failed progression at any cell cycle checkpoint proceeds preferentially through apoptosis and corresponds with the regulated removal of cells as a core biological mechanism (Figure 3).

Several proteins controlling cell cycle arrest are also capable of inducing apoptosis. The transcription factor Myc is best known as a potent inducer of proliferation through activating gene expression of cyclins A and D and downregulating p21 [115]. Aberrant Myc expression is connected to apoptosis by downregulation of antiapoptotic BCL-2 family members and activation of BAX [116,117]. Human tumor protein p53 (TP53) is a multifunctional protein that modulates the transcription of over 500 genes in response to an array of damaging stimuli. TP53 roles are well defined, including in cell cycle arrest and apoptosis through transcriptional regulation of sensitizing (PUMA, NOXA) and pore-forming (BAX) BCL-2 family members, death receptors such as Fas, and survival signaling (PTEN, Sestrins) [118,119]. Another example is Retinoblastoma protein (Rb), a multifunctional pocket protein family member that binds over 100 partners [120]. Described as binding and thus repressing genes encoding the E2F proteins, it is not surprising that Rb has other functional roles, including apoptosis. Contradictory evidence exists for Rb promoting cell survival through interactions with antiapoptotic BCL-2 members and as a target of effector caspases [121,122,123], as well as directly activating BAX and mitochondria driven apoptosis [124]. Further understanding of Rb protein-protein interactions is needed, but their nonnuclear activities are clearly intertwined with apoptotic pathways. Cell cycle progression is coordinated by the cyclic expression of cyclins and resultant activity of CDKs. These proteins are also involved in apoptosis, although specific functions have been challenging to elucidate and are likely context-dependent. Anti- and pro-apoptotic roles (often with overaccumulation) are described, as well as cleavage by caspases to prevent cell cycle events while committing to apoptosis [125,126].

Given that cell cycle regulation and apoptosis are intimately associated, the question remains of how a given stimulus or cellular response drives one pathway versus the other. This is an active area of research across many fields with no clear answers at present. Surely cellular context plays a role, as a response to the same damaging agent can be different across discrete cell types presumably due to genetic background, the status of other signaling pathways, tissue function, etc. Still, other factors such as modifications to the stimulus detecting proteins or secondary messengers, subcellular distribution, or epigenetic structure of target genes may contribute to cell fate. A plausible model is that the magnitude and duration of the activating signal influence whether a cell will (1) trigger initial cell cycle arrest while attempting to resolve the damage or (2) directly incite apoptosis [118]. This model often details a temporal aspect of the cell cycle and apoptosis relationship where failure to resolve the reason for arrest progresses to apoptosis.

### 3.2. Autophagy-Dependent Cell Death

Autophagy is a critical catabolic program that maintains homeostasis through the removal of defective organelles and proteins while also ensuring the availability of vital nutrient intermediates [127]. Dysregulation is known to be involved in a number of pathologies, including neurodegenerative disorders, cancer, and infectious diseases, underscoring the essential role of autophagy in cellular homeostasis [127]. While evidence of autophagy is often observed with other forms of cell death (presumably as a failed attempt to mediate survival), it has recently been appreciated that autophagic processes can also drive cell death directly [8,10]. It is now evident that autophagy-dependent cell death can proceed by two distinct pathways. Autosis, mentioned earlier, revolves around a membrane ATPase. A second pathway is exclusively reliant on core autophagy machinery and mechanisms (review [127]) and results in excessive auto-consumption and breakdown of endomembranes. Cell death by excessive autophagic flux does not appear to cause disruption of the plasma membrane [128,129], but this needs to be investigated directly to determine whether this is a characteristic of ADCD in all settings [130].

#### Cell Cycle Implications

Teasing apart autophagy and cell cycle programs are challenging due to the central involvement of mTOR in lysosomal modulation as well as cell growth and metabolism. Certainly, there are stressors, especially nutrient deprivation, that can trigger cell cycle arrest and autophagy in an attempt for cell survival. One mechanistic example is p27, with a canonical role as a pan-inhibitor of active CDKs to induce cell cycle while also capable of promoting autophagy via lysosomal recruitment and impeding mTORC1 activity, thereby enabling transcription factor EB promotion of lysosomal component biogenesis and autophagic activity [131]. It stands to reason that autophagic flux works reciprocally to cell cycle progression and often aligns with cell cycle arrest to triage resources for resolving arrest while maintaining core biological programs. Experimental evidence for this phenomenon is present, but this is in conflict with other data, possibly due to different experimental approaches and readouts of autophagy. This body of literature was recently reviewed, reaching a consensus that macroautophagy is suppressed by active CDKs to basal activity levels throughout the cell cycle, while CDK inhibitors are able to coordinately activate macroautophagy to support adaptation to the cellular environment [132]. In addition to CDK inhibitors, other kinases responsible for cell cycle arrests, such as ATM, ATR, and Chk1, have also been shown to promote autophagy [133,134,135]. If a stressor is insurmountable during cell cycle arrest, autophagic flux can be ‘switched off’ and transitioned to apoptosis through increased expression of proapoptotic proteins and decreased IAP family protein levels [136]. However, it may be speculated that inhibited or deficient apoptotic programs during cell cycle arrest allow prolonged autophagic flux that could escalate to autophagy-dependent cell death (Figure 3).

### 3.3. Efferocytosis

Apoptotic cells/bodies are scarce in healthy tissues due to highly efficient clearance and digestion by phagocytic cells, a process called efferocytosis. When apoptotic cells are not cleared, they degrade to secondary necrosis and lose their membrane integrity, spilling cellular contents (DAMPS) that elicit immune activation. Secondary necrosis is similar to primary necrosis (albeit with modified cellular contents due to initial apoptotic processing), meaning efferocytosis is an essential final step in non-immunogenic death and is important for homeostasis, tissue repair, and disease [137,138]. Efferocytosis is primarily carried out by professional phagocytes and relies on phagocyte targeting of processed cells via recognition of ‘eat me’ signals and bridging of cell surface receptors, such as TAM receptors [79,139]. Phosphatidylserine externalization on the plasma membrane is the preeminent biochemical event enabling immune cell recognition for efferocytosis and is intimately linked to apoptotic progression via caspase cleavage of flippase and cytochrome c oxidation of phosphatidylserine [140,141]. Efferocytosis research has primarily focused on apoptotic cell clearance but would also be necessary for the non-immunogenic removal of cells that die by heightened autophagic flux.

### 3.4. The Immune System and Cell Death

Cell death is a fundamental aspect of immunity both for the regulation of cellular-mediated immunity and the elimination of noxious cells. During lymphocyte development, a massive number of cells are produced to encompass broad antigen recognition capability, but the vast majority are eliminated as the immune response is whittled down to recognize specific antigens. Lymphocytes that do not form productive antigen recognition die by neglect, while lymphocytes that bind self-antigens with high affinities are selectively eliminated. In both cases, cell death is carried out by apoptotic mechanisms, with intrinsic and extrinsic pathways playing a role [26]. Clearance by apoptosis makes sense as cell elimination is a canonical part of lymphocyte development and does not merit further immune activation.

Licensed effector immune cells, mainly NK and cytotoxic T cells, eliminate foreign or infected cells through death receptor engagement and can elicit cell death by extrinsic apoptosis or through perforin and granzyme release upon receptor engagement. In the latter, perforins physically distort target cell membranes to permit granzyme entry. Granzymes are serine proteases that non-selectively cleave a myriad of proteins, including effector procaspases and antiapoptotic Bcl-2 proteins, to drive apoptosis, as well as many proteins downstream of caspases (such as histones, microtubules, and ICAD), thereby circumventing reliance on caspases for efficient elimination of targeted cells [142,143,144,145,146]. While immune effector cells primarily induce apoptosis in target cells, they can also contribute to immunogenic settings through cytokine production or upon aberrant perforin/granzyme activity.

An additional component of immunity is the opsonization of impaired cells and bacteria by antibody and complement proteins. Opsonins bridge to immune effector cells to facilitate the elimination of target cells by perforin/granzyme activity (NK and cytotoxic T cells) or phagocytosis and degradation of cellular components in a lysosomal process reminiscent of autophagy (phagocytes) [147,148]. Complement proteins can also directly kill cells in an immunogenic fashion through progressive deposition of complement proteins leading to the formation of a membrane attack complex. The complement membrane attack complex creates pores in the plasma membrane (of a similar structure to perforin pores), leading to unregulated movement of water and ions across the cell and eventual osmotic lysis [26,149].

## 4. Discussion

Regulated cell death proceeds through several pathways that are becoming increasingly understood. Death programs can be classified as immunogenic or not based on the breakdown or maintenance of plasma membrane integrity. An important consideration for understanding the various routes to cell death is that significant crosstalk exists among these pathways (see reviews [8,39,60,150,151]) with key nodes or molecules (such as inflammasomes, PANoptosome, Caspase-8, free Ca^++^, ROS) influencing multiple pathways. Evident from crosstalk relations is that non-immunogenic death pathways (apoptosis, ADCD) can progress to immunogenic death if the initial death program does not occur rapidly or the processed cell is not cleared before decaying to secondary necrosis. However, there is little evidence that the reverse can occur, with immunogenic death programs diverting to non-immunogenic death programs, even prior to plasma membrane disruption. Accordingly, immunogenic death pathways are carried out selectively, with varied susceptibility and core protein expression across cell types. For instance, pyroptosis is seen mostly in myeloid cells, while excitable cell types (muscle, neuronal) are predisposed to death pathways stemming from ion imbalances (e.g., mPTP-mediated necrosis). In most cases, activation leads to expression of the death pathway machinery, either de novo or via amplification loops, to ensure completion of cell death. Apoptotic proteins, on the other hand, are constitutively expressed, suggesting that all cells are intrinsically programmed to self-destruct. The expression of apoptotic proteins is also modulated through stimuli and other signaling pathways, but at steady-state, any cell can undergo apoptosis. If apoptosis is ubiquitously present, then cell survival relies on continual suppression of the apoptosis pathway rather than just promotion of survival pathways [1]. Sequestration of pore-forming BCL-2 proteins, inactive precursor caspases, and basal expression of inhibitors of caspases illustrate this phenomenon. Universal competence to apoptosis also clarifies why immune effector cells use this pathway to eliminate targeted cells.

Cancer is a highly lethal disease that exemplifies the suppression of death programs to support survival. A viable cancer cell not only requires physiological levels of apoptotic suppression but must also mitigate apoptosis triggers arising from hyperproliferative disease progression (e.g., lack of nutrients, compromised genomic integrity, elevated oxidative stress). Viewing cancer through the lens of cell death highlights the underlying developmental, and homeostatic relationship between apoptosis and the cell cycle and that proliferation is balanced predominantly through apoptosis. Understanding this relationship sheds new light on cancer treatment. The initial and still the most prominent strategies to treat cancer are chemotherapies that eliminate proliferative cells. This approach skews to eliminating cells that rapidly proliferate, evidenced by both significant anti-cancer activity and adverse effects from damage to high turnover tissues. However, there are two fundamental shortcomings with this strategy. First, by interfering with proliferation, i.e., the cell cycle, anti-proliferative agents rely on inducing cell cycle arrest, which progresses to apoptosis, or possibly autophagy-dependent cell death if apoptosis is sufficiently inhibited. Both death pathways have little to no consequence on the microenvironment and host immunity. Drug development strategies such as cell-permeable agents or improving tumor penetration attempt to circumvent these issues, but the fact remains that inducing apoptosis necessitates lethal drug activity in every cancer cell. In addition, there is accumulating data that apoptosis in a subpopulation of cells may actually support proliferation through processing and release of paracrine growth factors, modulation of local immune cells, and extracellular vesicle release (evolutionarily conserved mechanism of wound healing) [152,153,154]. Second, the obvious link between cell cycle and apoptosis, and cancer’s trademark control over both, set the stage for evasion strategies modulating either program. It is axiomatic that attempting to manipulate one element of a dyad (proliferation) leaves an avenue for evasion through the other component (apoptosis). Again, drug development efforts have attempted to address this through strategies like targeting anti-apoptosis proteins and combination therapies. Intuitively, sufficient levels of both agents would need to be present at the same time to achieve cell killing. Furthermore, cancer cells can employ a non-proliferative transition state to effectively leave this paradigm altogether [3,155]. While advances in cancer research and biotechnology have yielded novel modalities for therapeutics, a majority still target the proliferation-apoptosis axis (Table 3). To overcome therapeutic resistance in cancer, treatment strategies must deviate from solely apoptotic mechanisms of action.

It is increasingly recognized that some antiproliferative drugs can also incite non-apoptotic death pathways (through crosstalk or off-target effects), or initial apoptosis can diverge to other death pathways. For the latter to occur, apoptosis is first compromised, e.g., through heightened intrinsic inhibition or depleted cellular energy stores, and then stress-induced signaling triggers alternate death pathways. One such trigger is ROS, which is known to be elevated from a multitude of stress stimuli, including chemotherapy. ROS can strengthen apoptotic signaling but also initiate pyroptosis, ferroptosis, mPT-driven necrosis, or NETosis depending on cell type and state. Secondary mechanisms of action have gained interest in the past decade with the concept of immunogenic cell death (ICD) when considering cancer therapeutics and antiviral immunity [162]. ICD is defined by timed cell surface alterations and release of soluble antigens and adjuvants, ensuing antigen presentation to adaptive immune cells, and consequent activation of the immune system against cancer neoantigens. Although the exact molecular events of ICD are poorly understood, resulting interactions with the immune system are thought to be critical for altering the extent and composition of immune cell infiltrates into a targeted cancer lesion. Furthermore, tumor-specific immunity is proposed as a critical determinant of the efficacy of antineoplastic therapies (even in the case of conventional cytotoxins), while failure to induce ICD can explain incomplete clearance of malignant cells and eventual therapeutic failure. Interestingly, ICD has been demonstrated in a subset of cancer drugs, some of which have considerable efficacy records, with no correlation to the primary mechanism of action [162]. Thus, it is difficult to discern whether ICD arises from bystander caspase cleavages, involvement of other death pathways beyond apoptosis, through cell death-independent signaling events or a combination of events. Hallmarks of ICD suggest that ICD can be unrelated to other death pathways yet also involves cellular disintegration. Efforts are underway to identify and characterize therapies capable of inducing ICD [162,163], but thus far, this approach only involves empirical evaluation of already developed therapies.

**Figure 4:**
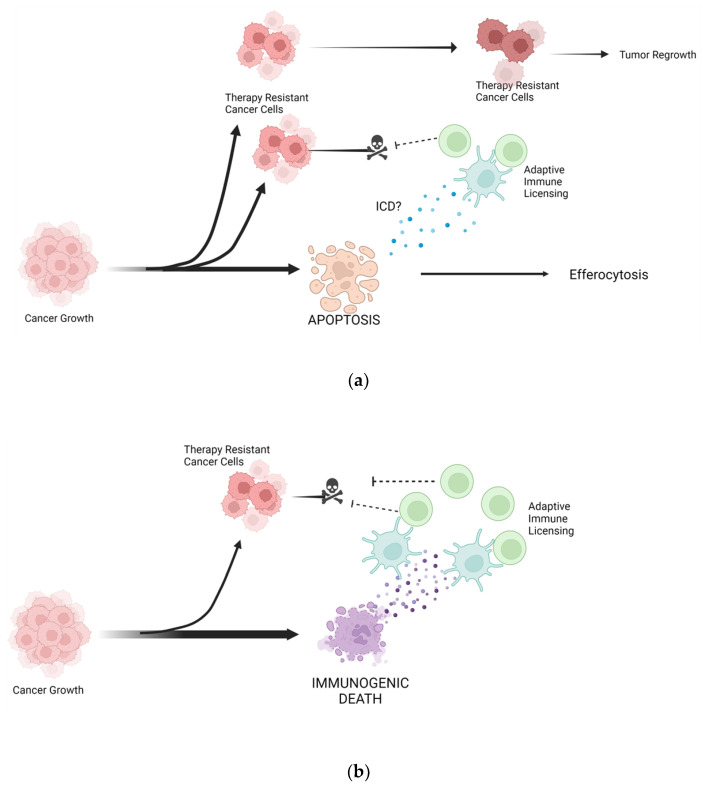
Engaging immunogenic death programs to circumvent therapeutic resistance. (**a**) Many cancer therapeutics are designed to elicit apoptosis, a physiological death program that cancer is inherently able to resist. In some cases, Immunogenic Cell Death (ICD) may develop during apoptosis and promote antitumor immunity, but this phenomenon is poorly understood. Therapy-resistant cancer is inevitable following conventional chemotherapy and eventually seeds relapse sites. (**b**) Immunogenic death pathways are cell cycle agnostic and selectively active across cell types. Thus, cancer is not evolutionarily equipped to evade these death pathways and may be less resistant to their induction. Additionally, therapeutics that elicit immunogenic death programs will efficiently license host immunity for auxiliary antitumor activity. Created with BioRender.com accessed on 21 April 2022.

ICD and rational combination with immunotherapy have established the capacity for immune system clearance of cancer, albeit in a minority of settings and with considerable variance in efficacy. An alternative or additional strategy to eradicate cancer is to design therapeutics that specifically engage immunogenic death pathways. Doing so would (1) manipulate cancer outside of the apoptosis, and cell cycle relationship and (2) directly activate, and possibly recruit, adaptive immunity (Figure 3 and Figure 4). Perturbance outside of pathways that cancer has evolved to co-manipulate plus licensing of host immunity represents a comprehensive therapeutic strategy. Understanding cell death mechanisms and analyzing the route to cell death will allow directed design toward immunogenic death programs. Targeting nonapoptotic cell death pathways will also provide much-needed insight into unexplored vulnerabilities of cancer. It is plausible that cancer’s reliance on hyperproliferation and apoptosis evasion creates susceptibilities to alternative forms of cell death. For example, caspase inactivation, ROS accumulation, heightened metabolic requirements, and elevated lipid content has all been documented in some cancer settings and is mentioned above as contributors to death pathways.

How a cancer cell dies and the resulting impact on the local microenvironment is an underappreciated aspect of cancer and long-term therapeutic failure. ICD is a prominent concept of study at present, but immunogenic death pathways are a largely overlooked alternative route to comprehensive anticancer immunity. Of course, a number of complications exist for this strategy as well, such as additional resistance mechanisms and pleiotropic effects of inflammation on cancer progression [164,165]. As cell death research continues and death pathways outside of apoptosis are used in therapeutic strategies, the field will gain valuable information on these limitations and different cancers susceptibilities. In the coming years, further understanding and appreciation of the diverse cell death pathways will refine distinctions between them and characterize their magnitude of immunogenicity in discrete settings. Many of the death pathways mentioned have critical gaps in knowledge and unexplored questions that need to be addressed when considering therapeutic strategies. For example, pyroptosis capacity in non-immune cells and induction of mPTP-mediated necrosis vs. mitochondrial apoptosis. An emphasis on elucidating structural organization and localization of pathway components (Box 1) will likely play an important role as the field utilizes diverse death programs in cancer therapy.

## Figures and Tables

**Table 2:** Core components of Non-Immunogenic Death Pathways.

	Apoptosis (Intrinsic)	Apoptosis (Extrinsic)	Autophagy-Dependent Cell Death
Key events	damage or stress stimuli MOMP caspase activity energy dependence	Death Receptor activation caspase activity energy dependence	stress or starvation states heightened autophagic flux
SMOC	Apoptosome (cytochrome c, Apaf-1, Caspase-9)	DISC (receptor DD, FADD, proCaspase-8)	n/a
effector functions	caspases cleave over 1000 substrates	caspases cleave over 1000 substrates	autophagosome accumulation breakdown of endomembranes maintenance of plasma membrane
cell cycle Propensity	activated following cell cycle arrest and mitotic death Active in any cell cycle phase	Active in any cell cycle phase	Active in any cell cycle phase Possible backup to apoptosis following cell cycle arrest

**Table 3:** Cancer therapeutics and cell death.

Common Modalities	Drug Examples	Primary Mechanism of Action	Primary Death Pathway
DNA damage	Platinum drugs, cyclophosphamides, anthracyclines, camptothecins, PBDs	damage DNA by direct binding, intercalation, or interactions with the topoisomerase family [5]	apoptosis
Microtubule dynamics	Taxanes, vinca alkaloids, auristatins	interfere with microtubule polymerization dynamics, disrupt mitosis [156]	apoptosis
Host immunity	CAR-T therapy, checkpoint inhibitors	exogenous engineered or endogenous T cell directed killing [26,142,143]	apoptosis
Antimetabolites	methotrexate, fluorouracil, gemcitabine	interfere with metabolism enzymes to disrupt metabolite levels [157]	apoptosis, ADCD
GFR antagonism	cetuximab, lapatinib, trastuzumab, sorafenib	inhibit growth factor binding or GFR activity attenuate proliferative signaling [158]	apoptosis
Proteasome inhibitors	bortezomib	block proteolytic subunits of proteasome, cause protein accumulation and unfolded protein response [159,160]	apoptosis
Monoclonal antibodies	cetuximab, trastuzumab, rituximab	bind target antigen and bridge to immune effector cells [161]	apoptosis

## Data Availability

Not applicable.
